# Allelopathic Potential of Aqueous Extract from *Acacia melanoxylon* R. Br. on *Lactuca sativa*

**DOI:** 10.3390/plants9091228

**Published:** 2020-09-18

**Authors:** M. Iftikhar Hussain, Mohamed A. El-Sheikh, Manuel J. Reigosa

**Affiliations:** 1 Department of Plant Biology and Soil Science, Faculty of Biology, University of Vigo, Campus Lagoas-Marcosende, E-36310 Vigo, Spain; mreigosa@uvigo.es; 2 CITACA, Agri-Food Research and Transfer Cluster, Campus da Auga, University of Vigo, 32004 Ourense, Spain; 3 Botany & Microbiology Department, College of Science, King Saud University, P.O. Box 2455, Riyadh 11451, Saudi Arabia; melsheikh@ksu.edu.sa

**Keywords:** allelopathic potential, chemical composition, phenolics, *Acacia melanoxylon*, *Lactuca sativa*, HPLC seedling growth Flavonoides

## Abstract

We studied the polyphenol (phenolic compounds and flavonoids) composition and allelopathic effects of *Acacia melanoxylon* R. Br. aerial foliage aqueous extract (0%, 25%, 50%, 75% and 100%) on the seedling growth and plant biomass of the general biotest species, lettuce *(Lactuca sativa*). Mean leaf fresh weight, leaf dry weight, root fresh weight and root dry weight were decreased following exposure to *Acacia* aerial foliage, flowers aqueous extract (AFE) and phyllodes aqueous extract (APE) after 6 days. The reduction in plant dry biomass was more than 50% following treatment with AFE. The decrease in mean root length was approximately 37.7% and 29.20% following treatment with *Acacia* flowers extract (AFE) at 75% and 100% concentration, respectively. Root dry weight of *L. sativa* was reduced by both flowers and phyllodes extract. The reduction of protein contents in lettuce leaves following *Acacia* foliage extract proved that both AFE and APE exhibit polyphenols that causes the toxicity which led to decrease in leaf protein contents. High-Performance Liquid Chromatography (HPLC) was employed to analyze the *A. melanoxylon* flowers and phyllodes. A total of 13 compounds (accounting for most abundant compounds in flowers and phyllodes) include different flavonoids and phenolic compounds. The phytochemical compounds detected were: Gallic acid, protocatechuic acid, *p*-hydroxybenzoic acid, *p*-hydroxybenzaldehyde, vanillic acid, syringic acid, *p*-coumaric acid, and ferulic acid. The major flavonoid compounds identified include rutin, luteolin, apigenin, and catechin. Allelopathic effects of flower and phyllodes extracts from *A. melanoxylon* may be due to the presence of above compounds identified by HPLC analysis.

## 1. Introduction

Allelopathy can be referred as “any process that involves secondary metabolites produced by plants, algae, bacteria and fungi that influence the growth and development of biological systems” [1]. For the last decades, the study of the allelopathic phenomenon has reached a specialized level of knowledge, but it is still necessary to introduce it into the modern ecophysiological concept to give answers to several unanswered questions [2,3]. The intensity of the allelopathic effect in the field will depend, between others, on the different transformations that the organic compounds will suffer after the release to the environment. Exotic plants are causing a serious threat to the native plants and ecosystem through interfering in growth and ecophysiological attributes of neighbours [4,5,6,7].

Nonindigenous plants present a serious risk to their neighboring plant [8]. Invasive plants possess several phytotoxic compounds, when released into the environment impede the germination and seedling growth of surrounding plant species at both ecosystem and species level [9,10]. Secondary metabolites are active allelopathic compounds released in the natural plant-soil-environment ecosystem from allelopathic crops, weeds, halophytes, shrubs and trees and their natural leachates might interfere with growth and physiological attributes of neighbouring plant species [3,10]. These toxic metabolites can be stored in the vacuole, polymerised or directly liberated, but anyway, they will be finally released to the environment where they can act as allelopathic agents on the metabolism of neighbouring plants, and giving usually an advantage to the producer [11].

*Acacia melanoxylon* R. Br. commonly known as Blackwood belonging to the family Fabaceae, subfamily Mimosoidae is a perennial tree, is native to Australia and Tasmania. It has now spread to various parts of the world including Asia, Africa and Europe [12]. The tree has wider ecological amplitude and has potential to grow in a wide range of soil types, and currently invading coastal habitats of North Western Iberian Peninsula, (Spain and Portugal). It has been listed as one of the most dangerous invasive species and currently invading the agriculture open fields, along the water channels, and rivers [13]. The allelopathic effects of *A. melanoxylon* on neighboring plants has been implied [14,15], but not thoroughly investigated. The phyllodes of *A. melanoxylon* have also been previously shown to contain allelochemicals [14] and affecting the natural vegetation [16]. The search for plant protection measures as an alternative to chemical weed control, which are widely used in agronomic and field and horticulture crops is of paramount importance [17,18].

Alternative control methods can take advantage of ecological processes such as using natural water extract of allelopathic plants, natural leachates and mixing the plant residues in the soil or applying allelopathic substances through foliar spray [3,19]. Several authors evaluated the role of allelopathy for weed management through allelopathic plant tissue aqueous extract. Brassica, mulberry and sorghum aqueous extracts have demonstrated their allelopathic potential in the laboratory and field experiments mainly through plant density reduction and biomass inhibition of *Cyperus rotundus* L. and *Trianthema portulacastrum* L. [20,21]. Allelopathic water extracts are water-soluble allelochemicals extracted from plants. Allelopathic water extract is used as natural herbicide because most of allelochemical compounds are water-soluble and are easy to apply without additional wetting agent, and they are more environmentally friendly than synthetic herbicide. According to Bajwa et al. [18], water extracts of mature fine grains greatly control the population and biomass of a weed competitor, and fine grain water extract reduces weed growth and density and increases wheat yield. However, allelochemicals from different plants and the concentration of the extract has different effects on the target plants, respectively [18].

Although, the role of *A. melanoxylon* allelopathy in controlling weeds and its phytotoxic impact is well known; nonetheless, influence of aerial foliage aqueous extract on the horticulture crops like lettuce has not been explored extensively. Meanwhile, as one of the most common cultivated vegetables in the open agriculture fields that are colonized by *Acacia melanoxylon* R. Br., lettuce’ seedlings are very sensitive to the changes in the environmental factorss and often used as a general biotest species in allelopathic bioassays [19]. The findings from this study will elaborate a solid foundation to better understand the allelopathic mechanism that drives the successful colonization of *A. melanoxylon* in the North Western Iberian Peninsula. These findings will also help policy makers to prepare guidelines and measures for prevention and possible control of invasive alien species. This study elucidated the three hypothesis: (I) *A. melanoxylon* foliage water extract recruit adverse influences on root/shoot length, fresh and dry weights of leaves and roots of lettuce and the adverse influences notably upsurge with increasing concentration of *Acacia* foliage extract; (II) flower and phyllodes aqueous extract intensify the allelopathy of *A. melanoxylon* on seedling growth of lettuce; (III) the allelopathic potential of *A. melanoxylon* foliage might be due to the presence of different polyphenols (phenolic compounds and flavonoids) and hence, these polyphenols can be used as lead compounds in herbicide discovery program.

## 2. Results

### 2.1. Effect of A. melanoxylon Foliage on Lettuce Growth and Biomass Accumulation

The phytotoxicity of *A. melanoxylon* (flowers and phyllodes) was evaluated by measuring the leaf and root length and biomass accumulation of *L. sativa* seedlings grown in the presence or absence of the *Acacia* aqueous extract. Table 1 and Table 2 show the mean leaf fresh weight (LFW), leaf dry weight (LDW), root fresh weight (RFW) and root dry weight (RDW) following exposure to *Acacia* aqueous extract (AAE) after 6 days. It can be seen that *Acacia* flower extract (AFE) (100%) significantly decreased the LFW (Table 1) while lettuce LFW was also reduced following treatment with *A. melanoxylon* phyllodes extract (Table 1). *Acacia* flower extract (AFE) exhibits a slight but significant phytotoxic effect on *L. sativa*, as revealed by a decrease in mean root fresh and root dry weight of approximately 51% and 42.5%, respectively, compared to the control (Table 2). Treatments, including AFE, are much more effective in reducing the leaf/root biomass, growth in *L. sativa*, with an inhibition percentage reaching more than 60% after treatment with *Acacia* aqueous extract aqueous extract (50%). There were non-significant differences in leaf dry/fresh weight ratio, root dry/fresh weight ratios, of the lettuce grown with different levels of *Acacia* flowers/phyllodes aqueous extract.

The shoot length of *L. sativa* significantly inhibited by *Acacia* phyllodes extract (APE) at each concentration tested, while 100% proved to be very lethal because it caused 50.78% phytotoxic effect in *L. sativa* shoot length (Table 3). The decrease in mean root length of was approximately 37.7% and 29.20% following treatment with *Acacia* flowers extract (AFE) (75% and 100%), respectively, compared to the control (Table 3). Treatments, including *Acacia* flowers extract (AFE), are much more effective in reducing the root growth in *L. sativa*, with an inhibition percentage reaching more than 50%.

The concentration-induced differences in root fresh weight that was significantly affected following treated with *Acacia* flowers extract (AFE) at each concentration tested are given in Table 4. Root dry weight of *L. sativa* was also reduced by both flowers and phyllodes extract (Table 4). However, AFE decreased the root dry weight upto 40% and 39% after treatment with 75% and 50%, respectively.

### 2.2. Effect of A. melanoxylon Foliage on Lettuce Leaf Protein Contents

**Figure 1:**
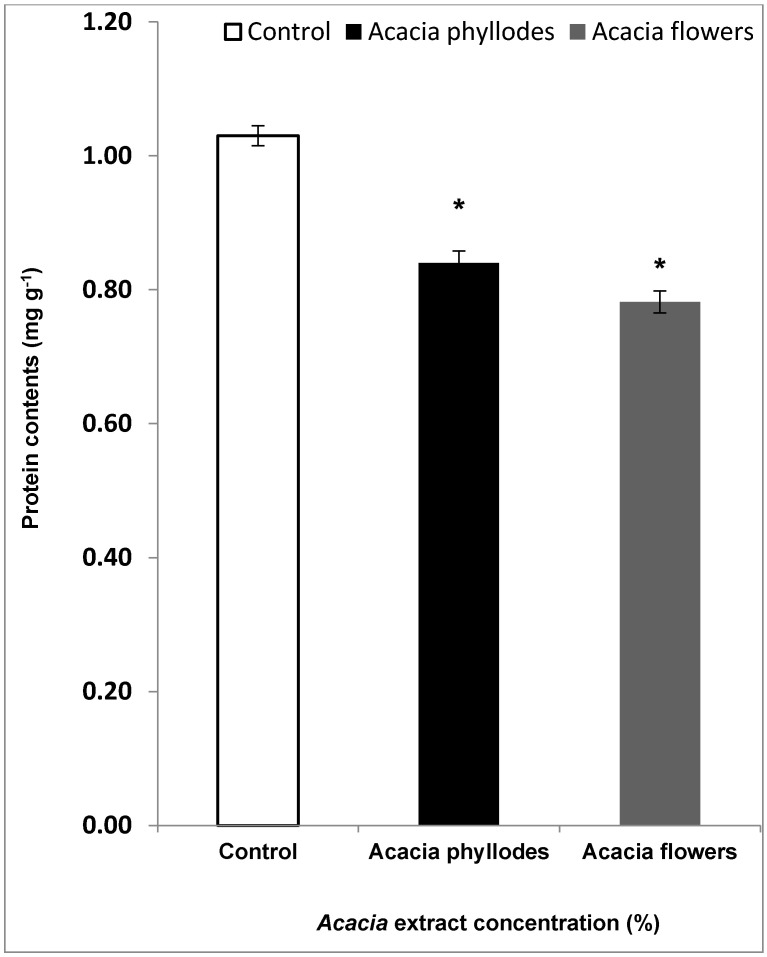
Inhibitory effects of *A. melanoxylon* flowers and phyllodes extract (0% and 100%) on the leaf protein contents (mg/g) of *Lactuca sativa*. Each bar represents the mean (± S.E.) of three replicates. * Asterisks indicate significant differences at level 0.05 with respect to the control.

Both AFE and APE decreased the protein contents of leaves, (0.78 mg g^−1^) and (0.83 mg g^−1^), respectively, following exposure at 100% as compared to control (1.03 mg g^−1^) (Figure 1). This reduction of leaf protein contents in lettuce leaves following *Acacia* aerial part extract proved that both AFE and APE exhibit polyphenols that causes the toxicity which led to decrease in leaf protein contents.

### 2.3. Chemical Composition of the Acacia melanoxylon Aerial Foliage

Table 5 demonstrtae the chemical composition of *Acacia melanoxylon* aerial foliage (flowers and phyllodes). Several phytochemical constituents were identified from foliage extracts but we concentrated mainly on phenolics and flavonoids. Among the phenolic compounds, eight (8) were most important and include Gallic acid, protocatechuic acid, *p*-hydroxybenzoic acid, *p*-hydroxybenzaldehyde, vanillic acid, syringic acid, *p*-coumaric acid, ferulic acid constituents.

Table 5 showed that *p*-hydroxybenzoic acid was in highest content (12.33 mg/L), followed by vanillic acid (9.7 mg/L) and were the principal compounds in the flowers and phyllodes extract. In the phyllodes methanol extract, the major flavonoid compounds were rutin, luteolin, apigenin, and catechin.

## 3. Discussion

Allelochemicals are released to the environment in different ways (by volatilisation, leaching, exudation or decomposition) and can act in a direct or an indirect way on the receptor plants [16,19,22,23]. Their chemical nature is complex and diverse (organic acids, aldehydes, coumarins, quinones, flavonoids, alkaloids, terpenoids, etc.), but the most part of them comes from three principal biosynthetic routes, the route of shikimic acid (benzoic and cinnamic acids and their derivatives, coumarins, glycosides, alkaloids, etc.), and the routes of acetic and mevalonic acids (terpenoids, steroids, complex quinones, etc.) [3,9,10,16]. Allelopathic compounds are present in almost all plants and they can be found in many parts of the plant like in roots, seeds, leave, fruits, stems, etc.

The leaf and root growth of lettuce was decreased following treatment with *Acacia melanoxylon* R.Br. aerial foliage aqueous extract at all concentration tested. The phytotoxicity of *A. melanoxylon* R.Br. was previously studied by Souto et al. [14], who reported that soil bioassays showed clear inhibitory effects on growth and germination of understory plants, particularly soils from *Eucalyptus* and *Acacia* stands. In this study, the effects of water soluble allelochemicals appear to alter a variety of morpho-physiological functions and significantly reduced the leaf fresh and dry biomass. Zhang et al. [24], showed that allelochemical (isoliquiritigenin) in the concentrations ranged between 0.2–1.0 mM exhibited toxic impacts that decreased the lettuce seedling growth. However, the results were dose dependent. They reported that isoliquiritigenin is an important phytochemical that caused >40% inhibition of radicle elongation in lettuce seedlings following exposure to 0.8 mM concentration. In another study, Sánchez-Moreiras et al. [25] documented that natural product 2-(3*H*)-benzoxazolinone caused a 50% reduction in the radicle length of lettuce at a concentration of 0.9 mM. Several authors indicated that it is extremely difficult to separate the growth and physiological secondary effects afrom the primary impacts caused by allelochemicals. However, the secondary impacts led to the disruption of cell differentiation, plant water status, water uptake, respiration, signal transduction, photosynthesis and enzyme function [3,6,7,10,17,26].

The germination and seedling growth (root, shoot length, plant biomass) of plant seeds constitutes a primary step in the growth and development of many plant species and demonstrate an importance to highlight the allelopathic activity. Our seedling growth bioassays showed that AFE and APE exhibit significant amount of water soluble allelochemicals and had phytotoxic impact (*p* > 0.05) on lettuce growth. These water soluble polyphenols (phenolic compounds and flavonoids) have allelopathic effect on seedling growth, biomass and biochemical traits (leaf protein contents) of lettuce. In some trials, low dose of allelochemicals showed stimulation while higher concentration was lethal. Similarly, Al-Wakeel et al. [27] documented that *A. nilotica* leaf extract enhanced the plant growth in peas, while a higher concentration was toxic that significantly decrease plant growth in the pea.

Several authors documented that, apart from environmental stresses, different invasive plant species showed phytotoxicity on the physiological and biochemical attributes of lettuce. In our results, *Acacia melanoxylon* flowers was highly deleterious at (100%) concentration tested and significantly inhibited protein contents in *L. sativa* as compared to control. However, Wang et al. [28] evaluated the leaf extract of alien invasive plant species, *Solidago canadensis* L. alone and joint application of nitrogen and cadmium stress. The *S.* *canadensis* employee distinctly allelopathy on germination and growth characteristics of lettuce and remarkably increased with increasing concentration of leaf extracts. However, N application relieved the allelopathy, while Cd treatment proved to be lethal, inhibiting the physiological traits of the lettuce.

In another study, BOA caused a significant reduction in leaf proteins due to protein denaturation, following exposure to allelochemicals. They reported that protein synthesis was significantly reduced following treatment with BOA [25]. However, contrary, there was an increase in leaf proteins in maize and decrease in kidney beans following treatment with *Acacia nilotica* extract [29]. Another study conducted by Lu et al. [30] showed that leaf extracts from three invasive plant species [(*Solidago canadensis* L., *Erigeron annuus* (L.) Pers., and *Conyza canadensis* L. Cronq)] inhibited the seed germination of lettuce. Meanwhile, heavy metals (Cu, Pb) promoted the invasion of all three plant species and allelopathic phenomena were more severe in the presence of heavy metals. Allelopathyic impact was significantly varied from one to other invasive species and *S. Canadensis* demonstrated moe phytotix than others. The allelopathic activity depended on a number of factors such as evaluating vegetation, extract types and concentration, and environmental attributes. Our results showed that aqueous extract of flowers and phyllodes from *A. melanoxylon* proved phytotoxic in nature (due to the presence of phytochemicals) and hence decreased the growth and morpho-physiological parameters of *L. sativa* in a dose-dependent manner (Table 1 and Table 2). These results are in conformity with the earlier reports of allelopathy in *A. melanoxylon* [13,14,15]. According to Chou et al. [31], foliage extract of *Acacia confusa* Merr showed significant inhibitory effects on target plant species. Similarly, different concentrations of *Acacia auriculiformis* A. Cunn. ex Benth. also showed phytotoxicity against a number of vegetations [32]. Similar results were reported for *Acacia nilotica* [29], *Acacia auriculiformis* [33], *Acacia nilotica* [27]. In the present study, we found that flowers of *A. melanoxylon* were more phytotoxic as compared to phyllodes. It might be due to the difference in phytotoxicity of different secondary metabolites present in two plant organs and their chemical structure. Several other allelopathic crops, such as sorghum root extract, significantly reduced growth and development of several weeds in the wheat fields [34]. The allelopathic crop residue also showed inhibition of germination of different weeds seeds [35].

The potential effect of the allelochemical will be higher or lower depending on the concentration, the soil transformations, and the target species, but also on the environmental factors and the simultaneous occurrence of other biotic or abiotic stresses. Most of the polyphenols, identified in the present study (Table 5) are assumed to be water soluble and, mostly, phenolics have good potential as templates for new herbicide classes [36,37]. Different polyphenols (phenolic compounds, flavonoids, derivatives of hydoxybenzoic acids, derivatives of cinnamic acids) were identified and seem to be water soluble; previously, it was elaborated that most of the allelochemicals, when in contact with plant cell walls, caused phytotoxicity and interfered in the ecophysiological parameters of the target plants [36,38,39]. These phytochemicals possess certain properties and can be used them as lead compounds for new herbicide discovery program [3,19,23,40,41].

In terms of the allelopathy bioassay employed in this study, it has been widely used in evaluating the allelopathic impact and is also short, simple and highly sensitive [42,43]. Germination, growth and seedling development conditions of the pot experiment are similar to those in the natural environment than that of the laboratory [30]. Therefore, it was suggested that growth and ecophysiological study of the target plant in the natural environment needs further investigation to study the allelopathic stress of the donor plant. Growth, fresh weight, dry weight, shoot length and root length attributes are important biological attributes that were selected as to evaluate the *A. melanoxylon* allelopathy, whose extract concentration will affect the abundance and competitiveness of recipient plants in natural habitat. However, at present, we have not studied the allelopathic effect of *A. melanoxylon* extracts on *L. sativa* seedlings at the molecular level, so the effects on cell division and elongation, growth regulation system and respiration need to be determined in the future context.

The polyphenol composition of *Acacia melanoxylon* aerial foliage is shown in Table 5. Several phytochemical constituents were identified from foliage extracts but we concentrated mainly on phenolics and flavonoids. Among the phenolic compounds, eight were the most important and include Gallic acid, protocatechuic acid, *p*-hydroxybenzoic acid, *p*-hydroxybenzaldehyde, vanillic acid, siringic acid, *p*-coumaric acid, and ferulic acid constituents.

The *p*-hydroxybenzoic acid was highest in content (12.33 mg/L), followed by vanillic acid (9.7 mg/L) and these were the principal compounds in the flower and phyllode extracts as shown in Table 5. In the phyllode methanol extract, major flavonoid compounds were rutin, luteolin, apigenin, and catechin. According to studies of Souto et al. (14), allelopathy was the main process involved in the inhibition of soil microbial activity and retard the germination attributes of *Lactuca sativa* L., *Dactylis glomerata* L. and *Trifolium repens* L. following *Acacia* and *Eucalyptus* population as compared to the autochthonous *Quercus robur* L. The phytotoxic effects were more prominent during *Acacia* blooming stages. In another investigation carried out through gas chromatography and mass spectroscopy, Freire et al. [44] documented the presence of several polyphenols and phytotoxic molecules such as cinnamic acid derivatives, spinasteryl glucoside and dihydrospinasteryl glucosides. Physiological and biochemical parameters (seedling growth, sugar contents and chlorophyll content) in *Zea mays* L. (maize) and *Phaseolus vulgaris* L. were decreased by *Acacia nilotica* and *Eucalyptus rostrata* leaf leachates (29). In a pot experiment, *Acacia nilotica* leaves extract showed a significant reduction in the growth and metabolic activities of 45-day-old pea (*Pisum sativum* L.) plants. Meanwhile, higher concentration was inhibitory while lower concentration was stimularory. Qualitative and quantitative HPLC analysis of water extract of *Acacia nilotica* leaves revealed that protocatechuic and caffeic acids were the principal phenolic compounds accompanied by ferulic, cinnamic acids and apigenin in higher quantity, whereas pyrogallic, *p-*coumaric, syringic acids and coumarin were found in trace amounts [27].

## 4. Methods and Materials

### 4.1. Plant Materials

The fresh aerial parts (shoots exhibiting flowers and phyllodes) of *Acacia melanoxylon* were gathered from natural population from the mountainous area, Lagoas Marcosende campus, University of Vigo, during blooming stage (Pontecedra province, Spain, 42.2406° N, 8.7207° W). The seeds of Lettuce (*Lactuca sativa* L. cv. Great Lakes California (Asteraceae, Asterales), a food and cash crop, were purchased from Semillas Fito (Barcelona, Spain) and used for seedling growth bioassays.

### 4.2. Extraction of Polyphenols and HPLC Analysis

The fresh plant tissue was cut into small pieces with scissors and placed in the laboratory at room temperature (25 °C) for air drying because phenolics are not stable to drying in an oven at high temperature. The dry flowers or leaves (1 g), were mixed with 50 mL of methanol/HCl (1000:1, *v*/*v*) in a 100-mL Erlenmeyer flask and kept in darkness for 12 h with hand shaking every three hours. The mixture was filtered by using a filter paper and a funnel. The filtrate was saved in refrigerator and the process was repeated with residual plant material for another 12 h by adding 50 mL of methanol/HCl. The mixture was filtered again and two methanolic fractions were combined (approx. 100 mL). This mixture was dried in rotary evaporator under vacuum. The temperature of rotary evaporator was maintained below 35 °C. The remaining material was dissolved in 40 mL of ethanol/water (2:8, *v*/*v*) and filtered. Afterwards, three sequential extractions were carried out with 20 mL of diethyl ether. The mixture was extracted with an extraction funnel by shaking vigorously for one minute each time, waiting until complete separation into two phases: the aqueous one in the lower part and an organic one in the upper part of funnel. The organic phases were removed and saved. We collected three ethereal phases in an Erlenmeyer flask. After that, three sequential extractions were carried out with 20 mL ethyl acetate on aqueous phase, obtaining three new organic phases that were collected and combined with ethereal ones. The total organic fractions obtained in this way were dehydrated with anhydrous sodium sulphate for 30 min to remove minimum residual water. Subsequently, it was filtered to withdraw sodium sulphate and evaporated to dryness in rotary evaporator. The final residue containing phenolics were re-dissolved in 1 mL methanol/water (1:1, *v*/*v*) and filtered through a 0.45-µm-pore-size nylon membrane filter and saved in refrigerator at 4 °C until HPLC analysis. The henolics were extracted by the method as proposed by Macheix et al. [45] and was optimized by Bolaño et al. [46].

### 4.3. Extraction of Flavonoids from Flowers and Leaves of A. melanoxylon

The flavonoids were extracted by the method of Markham 1989 with certain modifications. The fresh plant tissue was cut into small pieces with scissors and placed in the laboratory at room temperature (25 °C) for air drying. The dried and powdered flowers or leaves (10 g) were macerated with 300 mL of methanol/water (80:20) for 24 h at room temperature with continuous shaking. The extract was obtained through filtration by using a Buchner funnel with a filter paper. This mixture was dried in rotary evaporator under vacuum until all methanol was removed. The remaining material was re-dissolved in 40 mL of ethanol/water (2:8, *v*/*v*) and filtered. The resultant aqueous material was extracted with petroleum ether in an extraction funnel to remove fats, terpens, chlorophylls and xanthophylls. This extraction was repeated three times. Afterwards the mixture was extracted as per described in Phenolics acids, i.e., three sequential extractions with diethyl ether followed by three sequential extractions with ethyl acetate. The total organic fractions obtained in this way were dehydrated with anhydrous sodium sulphate for 30 min to remove minimum residual water. Subsequently, it was filtered to withdraw sodium sulphate and evaporated to dryness in rotary evaporator. The final residue was re-dissolved in 2.5 mL methanol and filtered through a 0.45 µm pore size nylon membrane filter and saved in refrigerator at −20 °C until HPLC analysis.

### 4.4. UV-DIODE ARRAY Chemical Analyses

Analysis was performed using a Shimadzu chromatograph equipped with a UV-DIODE ARRAY detector to identify phenolic and flavonoids. Identification of the compounds was made by using a reverse-phase Waters Nova-Pak C-18 (4.6 × 250 mm) column with a 4µm particle size. For flavonoids, the extracts were analyzed using two mobile phases: (A) methanol:phosphoric acid 999:l and (B) water: phosphoric acid 999:1. Linear gradients starting with 20% (A) and ending with 100% (A) were used over the first 40 min with an additional 5 min at 100% (A). The flow rate of the mobile phase was 1 mL/min and the eluate was analyzed at 250–400 nm. For the phenolic compounds, extracts were analyzed using two mobile phases: (A) water: acetic acid 98:2 and (B) water: methanol: acetic acid 68:30:2. Linear gradients starting with 100% (A) and ending with 20% (A) were used over the first 59 min, with an additional 6 min at 20% (A). The flow rate of the mobile phase was 0.8 mL/min and the eluate was analysed at 210–400 nm.

### 4.5. Acacia melanoxylon Flowers and Phyllodes Aqueous Extract Preparation for Bioassays

Fresh shoots of *Acacia melanoxylon* were collected from natural population in the surrounding area of Lagoas Marcosende campus of University of Vigo, during flowering period. The flowers and phyllodes were separated from branches and soaked in distilled water in the ratio of 1:1 (*w*/*v*) at room temperature and left in the laboratory for 24 h. Similar extraction techniques were used by Molina et al. [47], in their studies of *Eucalyptus* spp. and Lorenzo et al. [48], in their studies of allelopathic effects of *Acacia dealbata* L. The extract was collected, filtered through filter paper and described as 100%. Distilled water was added in this solution to make different dilution (75%, 50% and 25%). We examined effects of both phyllodes and flowers extracts made by mixing plant material in water, as opposed to the often criticized method of tissue disruption and organic solvent extraction that may yield artificially higher levels of specific compounds [37].

### 4.6. Plant Material and Growth Conditions

Lettuce (*Lactuca sativa* L. cv. Great Lakes California (Asteraceae, Asterales)), a food and cash crop and the most widely used model species in allelopathic studies [49], was selected because of its fast germination and homogeneity. The seeds of test species were purchased commercially from Semillas Fito (Barcelona, Spain). The seeds were placed in plastic trays (32 × 20 × 6 cm) with a 5-cm-deep layer of perlite (500 g/tray). The trays were irrigated on alternate days with tap water until germination of seeds and thereafter with 500 mL 1:1 Hoagland solution/tray, twice in a week. Seedlings were germinated in darkened at 20 °C temperatures in environmentally controlled growth chamber. For seedling growth, the environmental conditions were as follows; temperature: 18/8 °C (day/night) and 12/12 h (light/darkness) photoperiod, 80% relative humidity and 200 µmol m^−2^ s^−1^ irradiance. One month old seedlings (when plants have three fully expanded leaves), were transferred to pots (10 cm) containing perlite (70g) to stimulate the development of root system and shifted to the glass house with same growing condition and nutrient solution (100 mL/pot). Every second day the pots were well watered with tap water through an automatic irrigation system. One week later, treatment solutions (100 mL/pot) were applied three times (day one, three and five) and measurements were taken on each day. The temperature in the glass house was maintained at 21 ± 2 °C with a relative humidity of 75%. The glasshouse was ventilated with outside air to ensure steady CO_2_. Aqueous extract (100%, 75%, 50%, 25%) of *Acacia melanoxylon* (flowers and phyllodes) and control were watered three times (days 1, 3 and 5) with 100 mL solution. The experiment was laid out in Randomized Complete Block Design (RCBD) and replicated thrice.

### 4.7. Plant Growth Measurements

Information about plant height/root length was obtained with a ruler and values were expressed in cm. The fresh and oven dry plant leaves and roots weight were obtained by first weighting independently fresh leaves and roots then after drying these samples in a circulatory air oven at 70 °C for 72 h. The samples were weighed again to get dry weight of plant.

### 4.8. Statistical Analysis

Data was analyzed with the statistical package SPSS^®^ (version 21.00) for Windows^®^ (SPSS Inc., Chicago, IL, USA). Data were analysed by using one-way analysis of variance (ANOVA) (Sokal and Rohlf 1995) (when variance were homogeneous) or Kruskal-Wallis test (when heterogeneous). The LSD test as post hoc test was used to determine main differences between treatment means. Significant differences between means of treatment were compared at 5% probability level.

## 5. Conclusions

The exotic invasive tree in North Western Iberian Peninsula (Galicia, Spain), *Acacia melanoxylon*, their aerial foliage (flowers and phyllodes) extracts have negative effects on the growth and biomass of a general biotest species *Lactuca sativa* seedlings. Allelopathic effect of *A. melanoxylon* extracts at higher concentration was stronger than that of lower concentration and flowers extract showed stronger inhibition in different attributes than phyllodes extract. According to HPLC data of flowers and phyllodes extracts, several phyto-chemicals were identified and they are represented as derivatives of benzoic acid and cinnamic acid. Apart from these phenolic acids, a significant amount of flavonoids such as rutin, quercetin, luteolin, apigenin, and catechin were identified that enhanced the phytotoxicity of the extract. The presence of all these polyphenols thus affects the growth, biomass and root development rate of *L. sativa*. What’s more, the inhibitory effect of flower extract is stronger than that of phyllodes extract, which is one of the reasons that the allelopathic molecules are in much higher amounts in flowers than phyllodes in *A. melanoxylon*.

## Figures and Tables

**Table 1:** Effect of aqueous extract of aerial foliage of *Acacia melanoxylon* R. Br. on leaf fresh weight (g) and leaf dry weight (g) of one month old general biotest species, *Lactuca sativa* L. LDFW ratio (Leaf dry weight fresh weight ratio).

Growth Characteristics	Treatments	100%	75%	50%	25%
Leaf fresh weight (g)	Control	10.58 ± 0.60	10.58 ± 0.60	10.58 ± 0.60	10.58 ± 0.60
	Acacia phyllodes	7.73 ± 0.3 *	7.93 ± 0.5 *	8.63 ± 0.2 *	8.29 ± 1.0 *
	Acacia flowers	5.10 ± 0.4 *	5.62 ± 0.3 *	7.25 ± 0.34 *	6.85 ± 1.0 *
Leaf dry weight (g)	Control	1.63 ± 0.15	1.63 ± 0.15	1.63 ± 0.15	1.63 ± 0.15
	Acacia phyllodes	1.78 ± 0.04 *	1.77 ± 0.1 *	1.90 ± 0.4 *	1.98 ± 0.3 *
	Acacia flowers	1.23 ± 0.2 *	1.21 ± 0.4 *	1.45 ± 0.8 *	1.64 ± 0.2
LDFW ratio	Control	0.154 ± 0.07	0.154 ± 0.07	0.154 ± 0.07	0.154 ± 0.07
	Acacia phyllodes	0.230 ± 0.0 *	0.223 ± 0.00 *	0.221 ± 0.0 *	0.238 ± 0.05 *
	Acacia flowers	0.272 ± 0.1 *	0.215 ± 0.0 *	0.200 ± 0.0 *	0.240 ± 0.01 *

Each value represents the mean (± S.E.) of three replicates. * Asterik indicates significant differences as compared to control for *p* < 0.05 according to LSD test.

**Table 2:** Effect of aqueous extract of aerial foliage of *Acacia melanoxylon* R. Br. on on root fresh weight (g) and root dry weight (g) of one month old general biotest species, *Lactuca sativa* L. RDFW ratio (Root dry weight fresh weight ratio).

Growth Characteristics	Treatments	100%	75%	50%	25%
Root fresh weight (g)	Control	6.31 ± 1.1	6.31 ± 1.0	6.31 ± 1.1	6.31 ± 1.2
	Acacia phyllodes	4.57 ± 0.3 *	3.9 ± 0.6 *	4.11 ± 0.13 *	4.5 ± 0.4 *
	Acacia flowers	3.82 ± 0.1 *	3.44 ± 1.3 *	3.79 ± 1.3 *	3.07 ± 0.6 *
Rood dry weight (g)	Control	0.40 ± 0.02	0.40 ± 0.03	0.40 ± 0.04	0.40 ± 0.05
	Acacia phyllodes	0.27 ± 0.04 *	0.32 ± 0.01 *	0.29 ± 0.09 *	0.37 ± 0.04 *
	Acacia flowers	0.28 ± 0.2 *	0.28 ± 0.04 *	0.23 ± 0.001 *	0.27 ± 0.09 *
RDFW ratio	Control	0.064 ± 0.02	0.064 ± 0.02	0.064 ± 0.02	0.064 ± 0.02
	Acacia phyllodes	0.059 ± 0.01	0.082 ± 0.01 *	0.071 ± 0.01 *	0.082 ± 0.01 *
	Acacia flowers	0.073 ± 0.14 *	0.081 ± 0.01 *	0.061 ± 0.01 *	0.088 ± 0.01 *

Each value represents the mean (± S.E.) of three replicates. * Asterik indicates significant differences as compared to control for *p* < 0.05 according to LSD test.

**Table 3:** Effect of aqueous extract of aerial foliage of *Acacia melanoxylon* R. Br. on shoot length (cm) and root length (cm) of one month old general biotest species, *Lactuca sativa* L. RS ratio (Root shoot ratio).

Growth Characteristics	Treatments	100%	75%	50%	25%
Shoot length (cm)	Control	15.30 ± 0.23	15.30 ± 0.23	15.30 ± 0.23	15.30 ± 0.23
	Acacia phyllodes	7.53 ± 0.5 *	8.96 ± 1.5 *	7.66 ± 0.5 *	9.16 ± 1.6 *
	Acacia flowers	8.96 ± 2.2 *	8.0 ± 0.5 *	8.16 ± 2.0 *	9.66 ± 0.5 *
Root length (cm)	Control	27.77 ± 0.7	27.77 ± 0.7	27.77 ± 0.7	27.77 ± 0.7
	Acacia phyllodes	21.33 ± 2.3 *	19.0 ± 1.5 *	22.66 ± 2.5 *	21.5 ± 2.7 *
	Acacia flowers	19.66 ± 2.0 *	17.3 ± 2.0 *	22.0 ± 1.7 *	21.3 ± 2.0 *
RS ratio	Control	1.82 ± 0.03	1.82 ± 0.03	1.82 ± 0.03	1.82 ± 0.03
	Acacia phyllodes	2.83 ± 0.02 *	2.12 ± 0.02 *	2.95 ± 0.02 *	2.34 ± 0.02 *
	Acacia flowers	2.19 ± 0.01 *	2.16 ± 0.02 *	2.69 ± 0.01 *	2.20 ± 0.02 *

Each value represents the mean (± S.E.) of three replicates. * Asterisk indicate significant differences as compared to control for *p* < 0.05 according to LSD test.

**Table 4:** Effect of aqueous extract of aerial foliage of *Acacia melanoxylon* R. Br. on root fresh weight (g) and root dry weight (g) of one month old general biotest species, *Lactuca sativa* L.

Growth Characteristics	Treatments	100%	75%	50%	25%
Root fresh weight (g)	Control	6.31 ± 0.34	6.31 ± 0.34	6.31 ± 0.34	6.31 ± 0.34
	Acacia phyllodes	4.57 ± 0.7 *	3.90 ± 0.07 *	4.12 ± 0.3 *	4.49 ± 0.4 *
	Acacia flowers	3.82 ± 0.1 *	3.44 ± 0.29 *	3.79 ± 0.6 *	3.07 ± 0.6 *
Root dry weight (g)	Control	0.45 ± 0.042	0.45 ± 0.042	0.45 ± 0.042	0.45 ± 0.042
	Acacia phyllodes	0.27 ± 0.02 *	0.323 ± 0.04 *	0.290 ± 0.09 *	0.373 ± 0.04 *
	Acacia flowers	0.28 ± 0.06 *	0.286 ± 0.04 *	0.273 ± 0.01 *	0.270 ± 0.09 *
RDWFW ratio	Control	0.071 ± 0.02	0.071 ± 0.02	0.071 ± 0.02	0.071 ± 0.02
	Acacia phyllodes	0.059 ± 0.01 *	0.083 ± 0.01 *	0.070 ± 0.01ns	0.083 ± 0.01 *
	Acacia flowers	0.073 ± 0.04ns	0.083 ± 0.04 *	0.072 ± 0.01ns	0.088 ± 0.01 *

Each value represents the mean (± S.E.) of three replicates. * Asterisk indicate significant differences as compared to control for *p* < 0.05 according to LSD test.

**Table 5:** Phenolics and flavonoids found in flower and phyllode extracts of *Acacia melanoxylon* R. Br. RT: retention time (minutes) of compounds in column.

Sr. No.	Common Name	Scientific Name	Flowers mg/L	Phyllodes mg/L	RT
	PHENOLICS				
1	Gallic acid	3,4,5-trihydroxy benzoic acid	4.4	3.41	6.5
2	Protocatechuic acid	3,4-dihydroxybenzoic acid	5.06		12.9
3	*p*-Hydroxybenzoic acid	4-hydroxybenzoic acid	12.33	1.46	21.9
4	*p*-Hydroxybenzaldehyde		0.91	0.16	28.6
5	Vanillic acid	4-hydroxy-3-methoxybenzoic acid	9.7	1.64	32.2
6	Syringic acid	4-hydroxy-3,5-dimethoxybenzoic acid		2.64	40.1
7	*p*-Coumaric acid	4-hydroxycinnamic acid	3.19	3.6	49.7
8	Ferulic acid	4-hydroxy-3-methoxycinnamic acid	3.87		57.9
	FLAVONOIDS				
9	Rutin		5342.39	5032.87	20.4
10	Quercetin	3,3′,4′,5,7-Pentahydroxyflavone	326.4		25.2
11	Luteolin	3′,4′,5,7-Tetrahydroxyflavone	388.89	706.89	26.2
12	Apigenin	4′,5,7-Trihydroxyflavone		85.55	28.5
13	Catechin	(±)-3,3′,4′,5,7-Flavanpentol		765.44	7.9
